# Protective Effect of *Pediococcus pentosaceus* LI05 Against *Clostridium difficile* Infection in a Mouse Model

**DOI:** 10.3389/fmicb.2018.02396

**Published:** 2018-10-09

**Authors:** Qiaomai Xu, Silan Gu, Yunbo Chen, Jiazheng Quan, Longxian Lv, Dazhi Chen, Beiwen Zheng, Lichen Xu, Lanjuan Li

**Affiliations:** State Key Laboratory for Diagnosis and Treatment of Infectious Diseases, Collaborative Innovation Center for Diagnosis and Treatment of Infectious Diseases, The First Affiliated Hospital, School of Medicine, Zhejiang University, Hangzhou, China

**Keywords:** *Clostridium difficile*, probiotics, intestinal dysbiosis, tight junction proteins, inflammatory cytokines

## Abstract

*Clostridium difficile* infection (CDI) is a major cause of infectious diarrhea among hospitalized patients. Probiotics could be instrumental in restoring the intestinal dysbiosis caused by CDI. Here, we examined the protective effect of *Pediococcus pentosaceus* LI05 in a mouse CDI model. C57BL/6 mice were administrated *P. pentosaceus* LI05 (LI05 group) or sterile anaerobic PBS (CDI group) everyday for 14 days. Mice were exposed to antibiotics cocktail for 5 days; then challenged with *C. difficile* strain VPI10463. Mice were monitored daily for survival and weight loss. Colonic tissue and serum samples were assessed for intestinal histopathology, intestinal barrier function and systemic inflammation. The oral administration of *P. pentosaceus* LI05 improved the survival rate and alleviated the histopathological impact of *C. difficile*. Compared to the CDI group, the levels of inflammatory mediators in the colon as well as inflammatory cytokines and chemokines in serum were substantially attenuated in the LI05 group. *P. pentosaceus* LI05 alleviated the CDI-induced of disruption of ZO-1, occludin and claudin-1. Additionally, fecal microbiome analysis showed an enrichment in the abundance of the *Porphyromonadaceae* and *Rikenellaceae*, while, the relative abundance of *Enterobacteriaceae* were decreased. Our results demonstrated that the preventive effect of *P. pentosaceus* LI05 against CDI was mediated via improving tight junction proteins and down-regulating the inflammatory response. Therefore, *P. pentosaceus* LI05 could be a promising probiotic in CDI.

## Introduction

*Clostridium difficile* infection (CDI) imposes a considerable challenge to the healthcare systems worldwide (Lo Vecchio and Zacur, 2012). The past two decades have witnessed the emergence of hypervirulent strains, recurrence and an overall higher infection rates. Upon infection, patients can be presented with a variety of symptoms ranging from mild diarrhea to life-threatening conditions like pseudomembranous colitis, toxic megacolon and death (Leffler and Lamont, 2015). Risk factors for CDI include the use of broad spectrum antibiotics and older age (Bartlett et al., 1978; Chalmers et al., 2016). The pathogenic effects of *C. difficile* are mediated via two secreted toxins, toxins A and B which activates various inflammatory cytokines causing intestinal inflammation, which inactivate members of Rho GTPases, leading to neutrophilic colitis, loss of intestinal barrier function, and cell death (Leffler and Lamont, 2015). In the early 2000s, the emergency of a hypervirulent strain, NAP1/BI/027, secretes a third toxin (binary toxin, CDT), which may result in poor prognosis (O’Connor et al., 2009; Chitnis et al., 2013). Current mainstays for CDI treatment remains to be metronidazole, and vancomycin (Schaffler and Breitruck, 2018). The new treatment like fidaxomicin, showed an equivalent cure rate and a reduced risk of recurrence when compared with patients who receiving vancomycin in a study (Cornely et al., 2012). However, the emergence of novel outbreak-associated strains and the high recurrence rates urge clinicians and researchers to explore new therapeutic options.

Restoration of the intestinal microbiota and intestinal barrier after gut dysbiosis is crucial for the clearance of CDI (Mills et al., 2018; Valdes-Varela et al., 2018). A recent meta-analysis demonstrated that probiotics may play a vital role in preventing CDI (Shen et al., 2017). Specifically, the administration of probiotics within the first 2 days of the antibiotic course reduced the CDI risk by more than 50% among inpatients, and the efficacy of probiotics gradually decreased afterward (Shen et al., 2017). Consequently, maintenance of the gut flora balance through probiotics is crucial for the management of CDI (Shen et al., 2017; Valdes-Varela et al., 2018).

The use *Lactobacillus* species either alone or in combination has been intensively investigated for its therapeutic effect against CDI (Mills et al., 2018). Several evidence suggested an association between the absence of lactobacilli and the development of CDI among inpatients (Banerjee et al., 2009; Goldenberg et al., 2015; Ratsep et al., 2017; Mills et al., 2018). Further, lactobacilli could successfully antagonize the cytotoxic effects of *C. difficile* (Vanderhoof et al., 1999; Barker et al., 2017; Ratsep et al., 2017; Spinler et al., 2017). *Pediococcus pentosaceus* is another probiotic species that belongs to family *Lactobacillaceae*. Several strains of *P. pentosaceus* has been extensively used for food preservation and also they displayed remarkable probiotic properties including an antimicrobial activity and regulation of inflammation in animals (Lv et al., 2014b). *P. pentosaceus* LI05 (CGMCC 7049), isolated from the fecal samples of a healthy volunteer, demonstrated acid-tolerant and bile-tolerant traits and our previous results demonstrated a potentially protective effects against enteropathogens (Lv et al., 2014a,b; Shi et al., 2017). Taken together, in this study we aim to investigate the protective function of *P. pentosaceus* LI05 against *C. difficile* in a mouse model of CDI.

## Materials and Methods

### Strains and Culture Conditions

*Pediococcus pentosaceus* LI05 was cultured anaerobically in MRS broth (Oxoid, Thermo Fisher Biochemicals Ltd., Beijing, China) at 37°C for 24 h as detailed previously (Lv et al., 2014b). The cultures were centrifuged to pellet the cells for 10 min at 5000 × *g* and 4°C, washed with sterile phosphate buffer saline (PBS, pH 7.2) twice and resuspended in the same buffer. *C. difficile* strain VPI 10463 (ATCC 43255) active culture was inoculated into Difco cooked meat media (BD Diagnostic Systems, United States) and incubated for 36 h at 37°C under anaerobic conditions (80% N_2_, 10% CO_2_, and 10% H_2_) in an anaerobic workstation (AW300SG; Electrotek, England) (Chen et al., 2008). Next, the culture was centrifuged at 3200 × *g* for 10 min at 4°C. The pellets were washed twice in sterile PBS, re-suspended and administered intragastrically to the mice.

### Animals and Experimental Design

**FIGURE 1:**
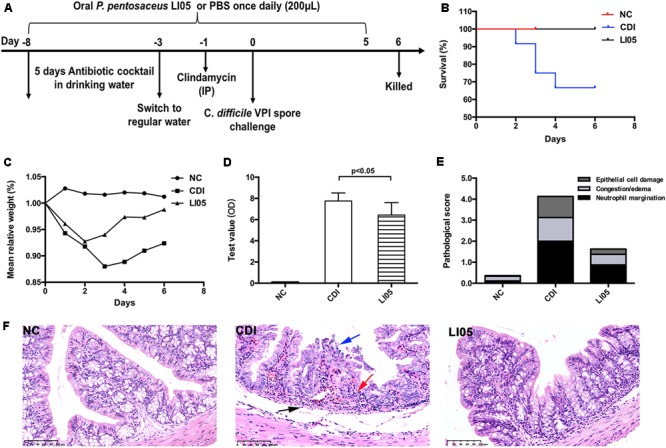
*Pediococcus pentosaceus* LI05 administration attenuated *C. difficile*-induced colonic tissue damage. **(A)** Schematic presentation of the experimental design. Mice were randomly assigned to three groups: NC group (*N* = 8), CDI group (*N* = 12), and LI05 group (*N* = 8). **(B)** Kaplan–Meier survival plots of three groups. **(C)** Mean relative weight of all surviving mice (up to the day of death). **(D)** Toxins A and B levels were measured in the fecal samples on day 3 and expressed as test value (ODs). **(E)** Pathologic score of colonic tissues. NC, normal control; CDI, *C. difficile* infected; LI05, *P. pentosaceus* LI05 treated. **(F)** Representative H&E staining of colonic tissues. The arrows showed epithelial damage (blue), neutrophil margination (red), and congestion/edema (black) (scale bar, 100 μm).

A total of 28 specific-pathogen-free (SPF) C57BL/6 female mice (6–8 week old) were acquired from Shanghai SLAC Laboratory Animal, Co., Ltd. Mice were allowed 1–2 weeks to acclimatize and were housed in groups of four per cage under SPF conditions. Next, mice were randomly divided into three groups based on the initial weight of mice: normal control group (NC group; *n* = 8), experimental model group (CDI group; *n* = 12), and *P. pentosaceus* LI05 group (LI05 group; *n* = 8). Using oral gavage, we administered a daily dosage of 3 × 10^9^ CFU *P. pentosaceus* LI05 suspended in 200 μl sterile PBS for 14 days in mice of the LI05 group. Probiotic administration started from the eigth day before *C. difficile* challenge till the fifth day post-infection (day -8 to day 5). In the NC and CDI groups, we administered 200 μl sterile anaerobic PBS once daily for 14 days. All mice were monitored at least once daily to observe the clinical symptoms of CDI, survival rate and record their body weight. The detailed experimental design is shown in **Figure 1**. All experiments were approved by the First Affiliated Hospital, School of Medicine, Zhejiang University’s institutional animal care and use committee and were performed in accordance with the recommendations of “Guide for the Care and Use of Laboratory Animals” (NIH publication 86-23 revised 1985).

### CDI Experimental Model

*Clostridium difficile* infection mouse model was established as detailed previously (Chen et al., 2008). Briefly, an antibiotic cocktail composed of kanamycin (0.4 mg/mL), gentamicin (0.035 mg/mL), colistin (850 U/mL), metronidazole (0.215 mg/mL), and vancomycin (0.045 mg/mL) was added to the drinking water for 5 days starting from the 8th day before CDI challenge to disrupt the normal microflora. Next, all mice were allowed access to regular drinking water for 2 days and received a single dose of clindamycin (10 mg/kg; intraperitoneal) 1 day prior to *C. difficile* challenge. After 24 h, mice in the CDI and LI05 groups were infected with 10^8^ CFU of *C. difficile* strain VPI 10463 by oral gavage. Stool samples were anaerobically cultured on cycloserine–cefoxitin–taurocholate agar (CCFA-TA; Oxoid) for 48 h as detailed previously (Chen et al., 2014; Xu et al., 2017). Matrix-assisted laser desorption ionization-time of flight (MALDI-TOF) mass spectrometry on a Microflex LT system (Bruker Daltonik) was used for strain identification. Further, toxins A and B were determined in triplicates by enzyme-linked fluorescent assay (VIDAS *C. difficile* toxins A and B, bioMerieux, SA) in the fecal samples according to the manufacturer’s instructions.

### Histological and Pathological Evaluation

Colon tissue samples were collected and fixed in 10% neutral buffered formalin. Samples were then embedded in paraffin and sliced into 4-μm thick sections on a microtome (RM2016, Leica, Shanghai). Next, samples were stained with haematoxylin and eosin (H&E) according to the standard protocols and analyzed by an experienced histopathologist in a blinded manner. The degree of enteritis was scored and graded using the scoring system reported by Chen et al. (2008). Briefly, histological scores were epithelial cell damage (score of 0–3), congestion/edema (score of 0–3), and neutrophil infiltration (score of 0–3).

### Immunohistochemical Staining and Immunofluorescence

Immunohistochemistry and immunofluorescence were performed in the colon tissues as previously reported (Lv et al., 2014a). Sections were stained with primary antibodies against (TNF-α, MCP-1, NF-KB p65, phospho-p65, and ZO-1) (Servicebio, Wuhan, China). Images were analyzed with the NanoZoomer Digital Pathology system (Hamamatsu Photonics K.K., Japan). A total of 10 vision fields were randomly selected from each slide and the mean positive area was calculated and analyzed by *Image J* software. The disruption and disorganization of ZO-1 immunostaining were visualized with a Zeiss LSM T-PMT confocal microscope (Zeiss, Jena, Germany).

### Detection of Serum Cytokines and Chemokines

Serum cytokine concentration was detected by Bio-Plex Pro Mouse Cytokine 23-Plex Panel (Bio-Rad) with a MAGPIX system (Luminex Corporation) and Bio-Plex Manager 6.1 software (Bio-Rad) according to the standard manufacturer’s protocol. The following cytokines and chemokines were included in the 23-plex panel: Eotaxin, G-CSF, GM-CSF, IL-1a, IL-1b, IL-2, IL-3, IL-4, IL-5, IL-6, IL-9, IL-10, IL-12(p40), IL-12(p70), IL-13, IL-17, IFN- γ, KC, MCP-1, MIP-1α, MIP-1β, RANTES, and TNF-α.

### Quantitative Real-Time PCR (qPCR)

Total RNA was extracted from the mice colons with RNeasy Plus Mini Kit (Qiagen, Hilden, Germany) according to the manufacturer’s protocols. RNA concentration was measured using a NanoDrop 2000 (Thermo Fisher Scientific, Waltham, MA, United States) and then reverse transcribed into cDNA with a PrimeScript^TM^ RT reagent Kit (Perfect Real Time) (RR036A, Takara, Dalian, China) following the standard protocols. The expression of mRNA was measured in triplicates with SYBR^®^ Premix Ex Taq^TM^ II master mix (Tli RNaseH Plus) (RR820A, Takara, Dalian, China) using a CFX96 Real-Time PCR Detection System (Bio-Rad). The relative expression of target genes was normalized to β-actin level and calculated by the comparative cycle threshold (Ct) method. Primer pairs were synthesized by Sangon Biotech Co., Ltd. (Shanghai, China) and listed in **Supplementary Table S1**.

### Analysis of Microbiota Composition by 16S rRNA Gene Amplicon Sequencing

Caecal content samples were snap frozen and stored at -80°C after collection. Bacterial DNA was isolated from the caecal contents using a QIAamp^®^ Fast DNA Stool Mini Kit (Qiagen, Hilden, Germany) following the manufacturer’s instructions. DNA concentration and integrity were measured by a NanoDrop 2000 spectrophotometer (Thermo Fisher Scientific, Hudson, NY, United States) and agarose gel electrophoresis, respectively. PCR amplification of the V3–V4 hypervariable regions of the bacterial 16S rRNA was carried out using universal primer pairs (343F: 5′-TACGGRAGGCAGCAG-3′; 798R: 5′-AGGGTATCTAATCCT-3′). The details of libraries construction are as follows: the libraries construction includes two stages, primers 343F and 798R were used in first stage PCR, the PCR system and conditions are: 95°C denaturation for 3 min, 25 cycles of 95°C denaturation for 30 s, 60°C annealing for 30 s, and 72°C extension for 30 s and a final extension for 10 min at 72°C. After the first stage PCR product was purified, primer i5 and i7 (Illumina universal adaptor) were used in second stage PCR and the PCR system and conditions: 95°C denaturation for 3 min, 8 cycles of 95°C denaturation for 30 s, 55°C annealing for 30 s, and 72°C extension for 30 s and a final extension for 10 min at 72°C. The second stage PCR product was purified using Agencourt AMPure XP beads (Beckman Coulter, Inc., Brea, CA, United States). Next, Sequencing was performed on an Illumina Miseq with two paired-end read cycles of 300 bases each (Illumina Inc., San Diego, CA, United States).

Paired-end reads were preprocessed using Trimmomatic software (Bolger et al., 2014) to detect and cut off ambiguous bases(N). It also cut off low quality sequences with average quality score below 20 using sliding window trimming approach. After trimming, paired-end reads were assembled using FLASH software (Magoc and Salzberg, 2011). Parameters of assembly were: 10 bp of minimal overlapping, 200 bp of maximum overlapping and 20% of maximum mismatch rate. Sequences were performed further denoising as follows: reads with ambiguous, homologous sequences or below 200 bp were abandoned. Reads with 75% of bases above Q20 were retained. Then, reads with chimera were detected and removed. These two steps were achieved using QIIME software (version 1.8.0) (Caporaso et al., 2010). Clean reads were clustered to generate operational taxonomic units (OTUs) using UPARSE software with 97% similarity cutoff (Edgar, 2013). The representative read of each OTU was selected using QIIME package. All representative reads were annotated and blasted against Silva database (Version 123) using RDP classifier (confidence threshold was 70%) (Wang et al., 2007). The microbial diversity in caecal content samples was estimated using the Chao1 index (Chao and Bunge, 2002). The UniFrac distance matrix performed by QIIME software was used for unweighted and weighted UniFrac Principal coordinates analysis (PCoA) and phylogenetic tree construction.

### PICRUSt Analysis

The functional profile of KEGG Orthology (KOs) (Kanehisa et al., 2012) for each sample was predicted from 16S rRNA sequence data with PICRUSt (Phylogenetic Investigation of Communities by Reconstruction of Unobserved States) (Langille et al., 2013). The predicted KO abundances were collapsed to level 3 by grouping them into a higher level of functional categorization.

### Statistical Analysis

Data were either expressed as mean ± standard deviation (SD) or median with interquartile range (IQR). One-way ANOVA test (for those with normal distribution) or Mann–Whitney’s *U* test (for those with skewed distribution) were used to compare the examine groups. To compare the differences in taxa abundances among the three groups, we utilized Wilcoxon rank sum tests and the Benjamini-Hochberg correction to reduce false discoveries for multiple hypotheses at each taxonomic level. The permutational multivariate analysis of variance (PERMANOVA) with the adonis function was used to test for microbial community clustering using weighted and unweighted UniFrac distance matrices. Statistical analyses were completed with either SPSS (22.0; SPSS Inc., Chicago, IL, United States) or GraphPad Prism (7; GraphPad Software Inc., San Diego, CA, United States). Two-sided *p*-values <0.05 were considered to be statistically significant.

## Results

### *P. pentosaceus* LI05 Reduced the Rate of Mortality and Protected Against CDI-Induced Weight Loss

In order to closely simulate the clinical status of CDI patients, we administered *P. pentosaceus* LI05 in the LI05 group on the first day of antibiotic cocktail use. Following CDI challenge, the survival rate of the NC, CDI, and LI05 groups were 100% (8/8), 66.67% (8/12), 100% (8/8), respectively (**Figure 1B**). The change in body weight acts as an indicator for the mice general health during CDI (Chen et al., 2008). Compared to the NC group, mice in the CDI group showed a significant weight loss following CDI and their body weights were at the lowest level on day 3 post-infection (CDI group: 88.00 ± 2.25 vs. NC group: 101.59 ± 2.6, *P* < 0.001; **Figure 1C**). Further, four mice in the CDI group were found dead by post-infection day 4. All mice became severely infected and moribund were humanely killed. In the LI05 group, mice suffered from a small decrease in body weight and all mice survived until the end of the experiment (**Figures 1B,C**). Compared to the CDI group, the mean relative weight loss of mice in the LI05 group was significantly lower on day 3 of (LI05 group: 94.01 ± 4.59 vs. CDI group, *P* < 0.01). In order to validate the responsibility of *C. difficile* to the observed CDI symptoms, we collected and analyzed stool samples before and after the *C. difficile* challenge. Stool samples collected before the challenge were negative for *C. difficile* toxins. Whereas, analysis of stool samples collected on day 3 post-infection in CDI and LI05 groups were both positive for *C. difficile* culture and toxins (**Figure 1D**).

### *P. pentosaceus* LI05 Attenuated CD-Induced Colon Injury

Histological inspection of the colon tissues from the CDI group demonstrated extensive submucosal edema, mucosal proliferation with patchy epithelial necrosis and the wide distribution of inflammatory cells, predominantly neutrophils, was evident (**Figure 1F**). On the other hand, compared to CDI group, the degree of colon injuries was less severe in the LI05 group (**Figure 1F**). The pathological grading system revealed that mice of CDI group were presented with severe epithelial damage, neutrophil margination, and congestion/edema and the total score was significantly higher than the NC group (CDI group: 4.13 ± 1.55 vs. NC group: 0.36 ± 0.22; *P* < 0.001; **Figure 1E**; Chen et al., 2008). Whereas, the total score was significantly lower in the LI05 group compared to the CDI group (1.63 ± 0.92 vs. 4.13 ± 1.55, respectively; *P* < 0.01; **Figure 1E**).

### *P. pentosaceus* LI05 Alleviated Immune Reactions by Modulating Serum Inflammatory Cytokine

**FIGURE 2:**
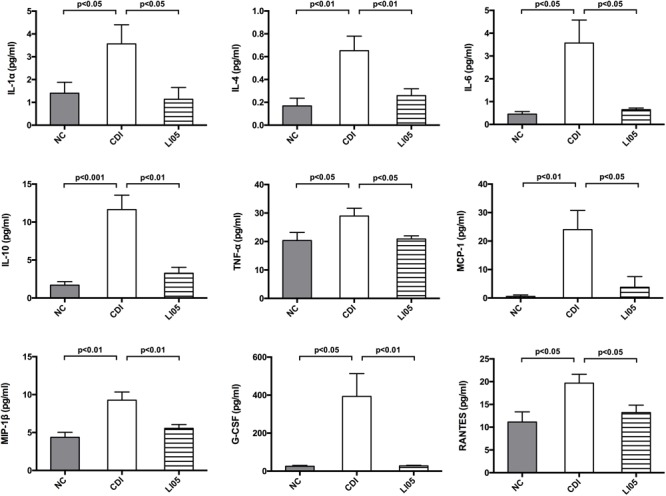
Administration of *P. pentosaceus* LI05 relieved *C. difficile*-induced cytokine expression in the serum. Bar charts represented the expression of IL-1α, IL-4, IL-6, IL-10 levels as well as the MIP-1β, MCP-1, G-CSF, TNF-α, and RANTES levels among the examined groups (*N* = 8 per group). NC, normal control; CDI, *C. difficile* infected; LI05, *P. pentosaceus* LI05 treated.

*Clostridium difficile* infection is aggravated through a broad range of inflammatory cytokines (Pawlowski et al., 2010). Therefore, we assessed profiles of 23 different serum cytokines to detect the impact of *P. pentosaceus* LI05 on the production of inflammatory mediators. The serum levels of the examined cytokines were significantly up-regulated in the CDI group compared to the NC group (**Figure 2**). In the LI05 group, nine serum cytokines were significantly mitigated (**Figure 2**). Therefore, these results indicate the *P. pentosaceus* LI05 could significantly improve the CDI-induced high interleukin levels (IL-1α, IL-4, IL-6) as well as the immunomodulatory cytokine IL-10. In addition, the increase in MIP-1β, MCP-1, G-CSF, TNF-α, and RANTES were also alleviated in the LI05 group (**Figure 2**).

### *P. pentosaceus* LI05 Mitigated CD-Induced Intestinal Immune Response and Reinforced Intestinal Barrier Integrity

**FIGURE 3:**
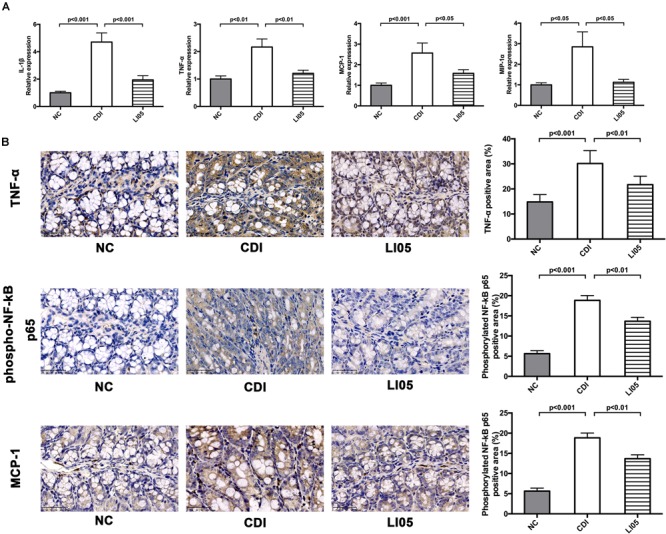
*Pediococcus pentosaceus* LI05 suppressed *C. difficile*-induced intestinal immune response. (*N* = 8 per group) **(A)** Colonic expression of the proinflammatory factors IL-1β, IL-4, TNF-α, MCP-1, and MIP-1α. **(B)** Immunohistochemical analysis of TNF-α, phospho-NF-κB p65 and MCP-1 in colonic tissue of mice (scale bar, 50 μm). Upper panel: Left, immunohistochemistry; Right, the percent positive area was illustrated by *Image J*. NC, normal control; CDI, *C. difficile* infected; LI05, *P. pentosaceus* LI05 treated.

Activation of NF-κB signaling pathway in monocytes and colonic epithelial cells is closely associated with intestinal inflammation due to *C. difficile* (Kim et al., 2006). Previous studies demonstrated that toxins secreted by *C. difficile* can activate the NF-κB pathway and subsequently a large number of proinflammatory factors are secreted leading to the colon inflammation (Cowardin et al., 2016). Therefore, we assessed the cytokine/chemokine mRNA expression in proximal colon among the three experimental groups. Compared to the NC group, mice in CDI group exhibited a significant increase in the relative mRNA levels of the following chemokines IL-1β, IL-4, TNF-α, MCP-1, and MIP-1α (**Figure 3A**). In contrast, mice in LI05 group exhibited a significant reduction of the above mentioned chemokines (*P* < 0.05). Furthermore, we investigated the impact of *P. pentosaceus* LI05 on NF-κB pathway via immunostaining the colon tissues with antibodies against TNF-α, MCP-1, and phospho-NF-κB p65 (**Figure 3B**). Compared to the CDI group, *Image J* analysis of the positive area percentage indicated that the levels of TNF-α, MCP-1, and phospho-NF-κB p65 were significantly lower in the LI05 group (30.16 ± 5.14 vs. 21.70 ± 3.38; 24.77 ± 3.73 vs. 21.70 ± 3.38, and 19.34 ± 3.38 vs. 13.68 ± 2.71, respectively; *P* < 0.01, *P* < 0.05, and *P* < 0.01, respectively).

**FIGURE 4:**
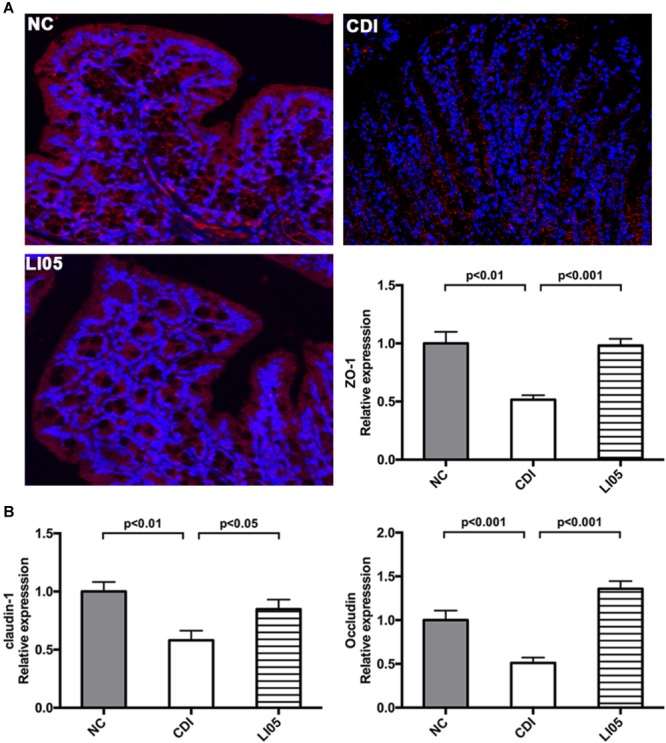
*Pediococcus pentosaceus* LI05 reinforced gut barrier function by mitigating the CD-induced disruption Tight Junction Proteins (ZO-1, occluding, claudin-1). (*N* = 8 per group) **(A)** ZO-1 immunofluorescence (×200) and mRNA expression of ZO-1 in colonic tissue. **(B)** mRNA expression of occluding, claudin-1 in colonic tissue. NC, normal control; CDI, *C. difficile* infected; LI05, *P. pentosaceus* LI05 treated.

Next, to assess the intestinal epithelial integrity, we examined the expression colonic intestinal tight junction proteins by quantitative PCR and immunofluorescence. In the CDI group, *C. difficile* challenge caused the delocalization of intestinal tight junction proteins (ZO-1, occludin, claudin-1) in the colons (**Figure 4**). In contrast, the LI05 group showed higher levels of ZO-1 mRNA expression and maintained high fluorescence intensities (**Figure 4A**). Notably, *P. pentosaceus* LI05 also promoted mRNA expression of occluding and claudin-1 compared to the CDI group (**Figure 4B**).

### *P. pentosaceus* LI05 Affected the CD-Induced Alterations of the Intestinal Microbiome and Metabolome

**FIGURE 5:**
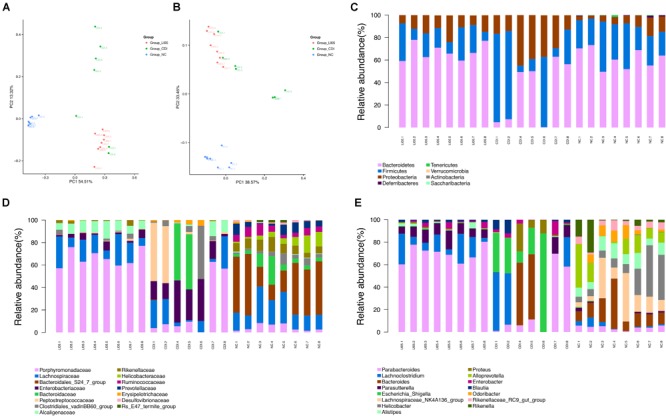
Administration of *P. pentosaceus* LI05 ameliorated microbiome dysbiosis in CDI. (*N* = 7–8 per group) **(A,B)** PCoA plot of the microbiota among three groups based on unweighted UniFrac metric **(A)** and weighted UniFrac metric **(B)**. Each point represented a sample. **(C)** Relative abundance of taxa at the phylum level. **(D)** Relative abundance of taxa at the family level. **(E)** Relative abundance of taxa at the genus level. NC, normal control; CDI, *C. difficile* infected; LI05, *P. pentosaceus* LI05 treated.

To further investigate the impact of *P. pentosaceus* LI05 intake on CDI mice, we assessed the fecal pellets by 16S rRNA sequencing. The sequencing data have been deposited in the NCBI SRA database with accession PRJNA490589. Fecal samples were obtained from 23 samples (NC group = 8, CDI group = 7, LI05 group = 8). 922,521 paired-end reads were generated, and 718,403 sequences (31235 reads per sample) were available for down-stream analysis after data processing and quality control. Species diversity calculated by Simpson diversity indices and the community richness detected by Chao1 indices indicated there was no significant difference between the CDI group and LI05 group (*P* = 0.119, *P* = 0.186, respectively). Next, we used the unweighted (**Figure 5A**) and weighted (**Figure 5B**) UniFrac PCoA to investigate the overall structural changes of microbial communities, which showed a marked difference between the CDI group and the NC group. The permutational multivariate analysis of variance (PERMANOVA) with the adonis function also revealed significant difference between three groups (NC vs. CDI *P* = 0.001; LI05 vs. NC *P* = 0.001; LI05 group vs. CDI group *P* = 0.011). Therefore, these results showed that *P. pentosaceus* LI05 can affect the CD-induced gut microbiome alteration. Furthermore, it appears that the CDI group had far more within group variation than the NC or LI05 group, it may be associated with the individual ability to fight this disease of mice.

Further, we compared relative taxa abundance at the phylum and genus levels to characterize the phenotypic changes in the taxonomic composition in depth (**Figures 5C–E**). Wilcoxon rank sum test combined with the Benjamini-Hochberg method was applied to compare bacteria taxa, and significant association was considered below a FDR threshold of 0.05. Compared with that of the NC group, our results demonstrated the existence of higher variations in individual CDI group. The percentage standard deviation of the three major phyla *Bacteroidetes, Firmicutes*, and *Proteobacteria* were 61.68% ± 8.89, 29.16% ± 9.08, and 8.19% ± 4.88 for NC group, respectively, and 32.97% ± 27.47, 39.25% ± 33.30, and 27.67% ± 13.24 for CDI group, respectively (**Figure 5C**). However, there was no significant difference of the three major phyla between LI05 and NC group (**Figure 5C**). Families such as *Rikenellaceae* (NC group 8.13% ± 2.49 vs. CDI group 0.11% ± 0.20), *Ruminococcaceae* (NC group 5.93% ± 2.40 vs. CDI group 0.04% ± 0.056), *Bacteroidales* (NC group 35.45% ± 16.31 vs. CDI group 0.09% ± 0.23), and *Deferribacteraceae* (NC group 0.05% ± 0.06 vs. CDI group 0.00% ± 0.00) were relatively deficient in CDI group compared to the NC group (*q* < 0.05), whereas, *Enterobacteriaceae* (NC group 0.05% ± 0.07 vs. CDI group 20.25% ± 11.89) and *Enterococcaceae* (CDI group 0.26% ± 0.26 vs. NC group 0.003% ± 0.004) was abundantly higher in the CDI group (*q* < 0.05) (**Figure 5D**). Compared with that of the CDI group, the continuous administration of *P. pentosaceus* LI05 lead to the decrease of the proportion of phylum *Proteobacteria* (13.13% ± 5.21 in LI05 group, 27.67% ± 13.24 in CDI group, *q* < 0.05), and the enrichment of phylum *Bacteroidetes* abundance (67.44% ± 7.35 in LI05 group, 32.97% ± 27,47 in CDI group, *q* < 0.05). At the family level, the relative abundance of *Porphyromonadaceae* (LI05 group 65.89% ± 7.49 vs. CDI group 20.41% ± 26.73) and *Rikenellaceae* (LI05 group 1.46% ± 1.66 vs. CDI group 0.11% ± 0.20) in LI05 group compared to the CDI group (*q* < 0.05), while, the relative abundance of *Enterobacteriaceae* (LI05 group 3.86% ± 2.92 vs. CDI group 20.25% ± 11.89, *q* < 0.05) were decreased in LI05 group (**Figure 5D**).

**FIGURE 6:**
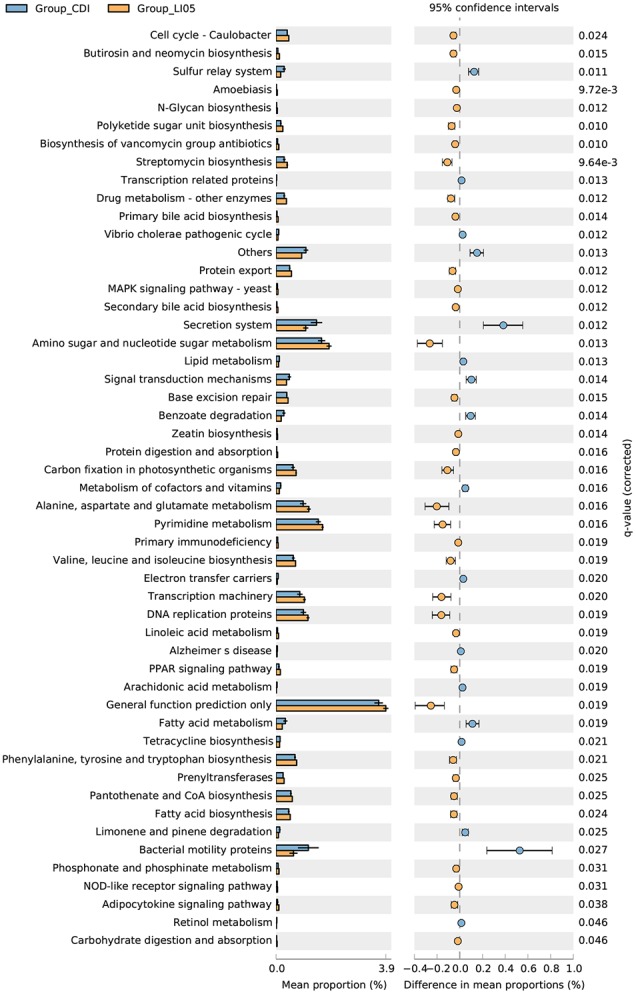
PICRUSt results of metabolic pathways in the gut microbiome of CDI group and LI05 group.

These compositional and diversity changes induced by *C. difficile* may have functional implications. We used PICRUSt, a bioinformatics tool that uses evolutionary modeling to predict metagenomic functional content from 16S rRNA data and a reference genome database, to assess potential functional differences among the three groups (Langille et al., 2013). Many of the predicted functional differences were in KEGG metabolic pathways between the microbiomes of CDI group and NC group: lipids, amino acids, peptides, carbohydrates, and xenobiotics (**Supplementary Figure S1**). However, the lipid metabolism pathway, Fatty acid metabolism and glycolytic pathway, including primary bile acid biosynthesis and secondary bile acid biosynthesis, was significantly different after administration of *P. pentosaceus* LI05 (**Figure 6**).

## Discussion

To the best of our knowledge, this is the first report that demonstrates the successful prophylactic function of *P. pentosaceus* against CDI. In this study, we observed that the administration of *P. pentosaceus* LI05 significantly reduced the mortality, substantially ameliorated CDI-induced colonic tissue damage and systemic inflammatory response in a mouse model treated with broad spectrum antibiotics and infected with *C. difficile* VPI 10463.

To date, the standard laboratory investigations cannot distinguish between *C. difficile* colonization and true CDI in the fecal samples (Bagdasarian et al., 2015). Further, previous studies of experimentally induced CDI focused on the inflammatory response in colonic tissues (Pawlowski et al., 2010; Koon et al., 2016; Roychowdhury et al., 2018). Nevertheless, information regarding the inflammatory mediators circulating in the blood is still limited. We expect that a better understanding for the inflammatory response in serum may highlight specific inflammatory pathways for CDI and hence, pave the road toward more efficient therapeutic options for CDI. Compared to the NC group, our results demonstrated that the serum inflammatory cytokines in the CDI group were significantly increased. The administration of *P. pentosaceus* LI05 significantly alleviated the CDI-induced inflammatory response. Similarly, in colon tissues the level of TNF-α, MCP-1, MIP-1α were up-regulated in the CDI group and *P. pentosaceus* LI05 treatment significantly improved the inflammatory response in the LI05 group. The up-regulation of serum cytokines often induces the production and secretion of G-CSF from the T cells, endothelial cells, and macrophages (Mehta et al., 2015). G-CSF plays an important role in promoting the production of granulocytes (Mehta et al., 2015). Once the infection is controlled the G-CSF levels are decreased and return back to normal. In the same context, MCP-1 and MIP-1α, belong to CC-chemokine family, play an important role in recruiting and activating monocytes/macrophages, regulating the inflammatory mediators and adhesion molecules (Carr et al., 1994; Kostova et al., 2015). Compared with the CDI group, the down-regulation of the abovementioned inflammatory mediators in LI05 group suggests that the clinical manifestations of the infection were drastically reduced in those mice.

*Clostridium difficile* pathogenicity is mainly attributed to the production of two exotoxins: toxins A and B (Di Bella et al., 2016). Both toxins share a common domain structure with around 63% of amino acid homology (von Eichel-Streiber et al., 1992; Pruitt et al., 2010) and have similar mechanisms in eliciting cell death (Cowardin et al., 2016). However, Ng et al. (2010) demonstrated that both toxins have remarkable differences in their pathogenic ability; specifically, toxin B is able to induce inflammasome activation at much lower concentrations than toxin A. Activation of NF-κB signaling pathway in colonic tissues was reported to be closely associated with toxin A (Kim et al., 2006; Cowardin et al., 2016). In turn, NF-κB phosphorylation induce the expression of pro-IL-1β gene and TNF-α (Cowardin et al., 2016). Further, challenging monocytes with *C. difficile* resulted in the induction of pro-inflammatory cytokines such as IL-1β and increased proliferation of allogenic T cells (Bianco et al., 2011; Vohra and Poxton, 2012). Increased TNF-α and IL-1β levels could further enhance toxins A and B-mediated cytotoxicity resulting in cell death (Cowardin et al., 2016). In our study, the administration of *P. pentosaceus* LI05 significantly reduced the inflammatory response in colonic tissues. Particularly, our results demonstrated that *P. pentosaceus* LI05 administration inhibited the NF-κB phosphorylation, decreased the intestinal inflammatory response, and down-regulated the expression of inflammatory genes and inflammatory cytokine, including TNF-α, MCP-1, IL-1β, MIP-1α in the LI05 group. As a result, we observed that the administration of *P. pentosaceus* LI05 improved the histological pictures of colonic tissues through decreasing the leukocyte infiltration, edema and epithelial necrosis compared to CDI group.

In agreement, previous studies demonstrated the crucial role of gut microbiota against CDI (Gu et al., 2016; Mills et al., 2018; Valdes-Varela et al., 2018). The restoration of intestinal microbiota and intestinal barrier after gut dysbiosis was found to be instrumental in CDI treatments (Mills et al., 2018; Valdes-Varela et al., 2018). Several antagonistic mechanisms have been proposed to explain the beneficial effects of probiotics including the production of antimicrobial molecules and modulation of intestinal inflammation (Boonma et al., 2014; Gebhart et al., 2015). Germination and growth of *C. difficile* were strongly affected by members of the two dominant phyla, *Firmicutes* and *Bacteroidetes* (Theriot et al., 2014, 2016; Antharam et al., 2016). The gut metabolome of CDI was associated with relative increases in amino acids, carbohydrates, primary bile acids, while levels of the secondary bile acid deoxycholate and luminal dipeptides decreased (Theriot et al., 2014). In summary, CDI in mice resulting in microbiome depletion, especially of *Bacteroidetes* species, decreased microbial diversity, and altered the potential functional capabilities of the microbiome. In our study, many of the predicted functional differences in KEGG metabolic pathways were found between LI05 group and CDI group, such as lipid metabolism pathway, fatty acid metabolism and glycolytic pathway. Furthermore, we previously verified that *P. pentosaceus* LI05 could synthesize three antimicrobials, including prebacteriocin, lysin, and colicin V production family proteins, and we verified their protective effects against tested enteropathogens (Lv et al., 2014b). Moreover, we also demonstrated that *P. pentosaceus* LI05 had the ability to accelerate the reproduction of generally beneficial microbial taxa like *Lactobacillus, Prevotella*, and *Paraprevotella* as well as restraining the excessive reproduction of opportunistic pathogens like *Oscillospira, Flavonifractor*, and *Escherichia* (Lv et al., 2014a,b; Shi et al., 2017). In good agreement, results obtained from this study showed *Escherichia* was significantly decreased in LI05 group, thus, we speculated that *P. pentosaceus* LI05 may inhibit the opportunistic pathogen like *Escherichia* in mice after exposure to broad spectrum antibiotics and *C. difficile* challenge. However, since we only sequenced partial 16S rRNA sequences in this study, and the OTUs could not be assigned to the species level with high confidence, *P. pentosaceus* LI05 was not observed to be enriched in the gut microbiome. This is likely because of no significant increase in the amount of *P. pentosaceus* LI05 in the mice caecal content samples in such a short time after probiotic withdrawal.

In adult Chinese patients, we previously reported that the percentages of *Escherichia*/*Shigella* were significantly higher in CDI patients (Gu et al., 2016). The overgrowth of *Escherichia*/*Shigella* could possible impair the intestinal permeability resulting in worsening disease severity and complications such as endotoxaemia (Quigley et al., 2013). Therefore, here, we investigated the integrity of the intestinal barrier to explore the mechanism of impaired intestinal permeability. In epithelial cells, toxins A and B were found to disrupt the tight junctions (Nusrat et al., 2001; Schenck et al., 2013). Here, we observed that CDI caused the delocalization of intestinal tight junction proteins (ZO-1, occluding, claudin-1). Upon the administration of *P. pentosaceus* LI05, that the intestinal tight junction protein (ZO-1, occludin, claudin-1) in the colon appeared more visually intact in the LI05 group. Taken together, in this study our results verified that the injured villi architecture and cytokine/chemokine was substantially ameliorated in the LI05 group. And our results also showed that *P. pentosaceus* LI05 can clearly affected the CD-induced gut microbiome alteration, thus, we hypothesized that it may be helpful in modulating gut barrier integrity. Nevertheless, one important limitation to this study is that the model of study is C57BL/6 mice. Although the mice model we used could closely represent the clinical manifestations and typical histologic features of this human disease (Chen et al., 2008). In this study, we showed the beneficial function of *P. pentosaceus* LI05 as a promising probiotic agent against CDI. These promising findings merit further experimental and clinical studies to determine the efficacy and safe applicability of *P. pentosaceus* LI05 in human subjects.

## Conclusion

In this study, we confirmed the protective role of *P. pentosaceus* LI05 against antibiotic cocktail treatment followed by CD challenge in mice. We suggest that *P. pentosaceus* LI05 pretreatment may reduce the risk of CDI by down-regulating the degree of intestinal inflammatory response and the production of serum inflammatory mediators, protecting the intestinal barrier function. These findings verify the beneficial function of *P. pentosaceus* LI05 as a promising probiotic agent against CDI.

## Author Contributions

LjL, QX, and SG designed the study. QX, SG, and JQ performed the experiments. YC, BZ, and LxL analyzed the data. QX, DC, and LX drafted the manuscript. LjL revised the paper. All authors contributed and approved the final article.

## Conflict of Interest Statement

The authors declare that the research was conducted in the absence of any commercial or financial relationships that could be construed as a potential conflict of interest. The reviewer CMB and handling Editor declared their shared affiliation.

## Supplementary Material

FIGURE S1 PICRUSt results of metabolic pathways in the gut microbiome of CDI group and NC group.

TABLE S1 PCR primer sets for RT-PCR analysis of the indicated genes.

## References

[B1] Antharam V. C. McEwen D. C. Garrett T. J. Dossey A. T. Li E. C. Kozlov A. N. (2016). An integrated metabolomic and microbiome analysis identified specific gut microbiota associated with fecal cholesterol and coprostanol in *Clostridium difficile* infection. *PLoS One* 11:e0148824. 10.1371/journal.pone.0148824 26871580 PMC4752508

[B2] Bagdasarian N. Rao K. Malani P. N. (2015). Diagnosis and treatment of *Clostridium difficile* in adults: a systematic review. *JAMA* 313 398–408. 10.1001/jama.2014.17103 25626036 PMC6561347

[B3] Banerjee P. Merkel G. J. Bhunia A. K. (2009). *Lactobacillus delbrueckii* ssp. *bulgaricus* B-30892 can inhibit cytotoxic effects and adhesion of pathogenic *Clostridium difficile* to Caco-2 cells. *Gut Pathog.* 1:8. 10.1186/1757-4749-1-8 19397787 PMC2680912

[B4] Barker A. K. Duster M. Valentine S. Hess T. Archbald-Pannone L. Guerrant R. (2017). A randomized controlled trial of probiotics for *Clostridium difficile* infection in adults (PICO). *J. Antimicrob. Chemother.* 72 3177–3180. 10.1093/jac/dkx254 28961980 PMC5890711

[B5] Bartlett J. G. Chang T. W. Gurwith M. Gorbach S. L. Onderdonk A. B. (1978). Antibiotic-associated pseudomembranous colitis due to toxin-producing clostridia. *N. Engl. J. Med.* 298 531–534. 10.1056/NEJM197803092981003 625309

[B6] Bianco M. Fedele G. Quattrini A. Spigaglia P. Barbanti F. Mastrantonio P. (2011). Immunomodulatory activities of surface-layer proteins obtained from epidemic and hypervirulent *Clostridium difficile* strains. *J. Med. Microbiol.* 60(Pt 8) 1162–1167. 10.1099/jmm.0.029694-0 21349985

[B7] Bolger A. M. Lohse M. Usadel B. (2014). Trimmomatic: a flexible trimmer for Illumina sequence data. *Bioinformatics* 30 2114–2120. 10.1093/bioinformatics/btu170 24695404 PMC4103590

[B8] Boonma P. Spinler J. K. Venable S. F. Versalovic J. Tumwasorn S. (2014). *Lactobacillus rhamnosus* L34 and *Lactobacillus casei* L39 suppress *Clostridium difficile*-induced IL-8 production by colonic epithelial cells. *BMC Microbiol.* 14:177. 10.1186/1471-2180-14-177 24989059 PMC4094603

[B9] Caporaso J. G. Kuczynski J. Stombaugh J. Bittinger K. Bushman F. D. Costello E. K. (2010). QIIME allows analysis of high-throughput community sequencing data. *Nat. Methods* 7 335–336. 10.1038/nmeth.f.303 20383131 PMC3156573

[B10] Carr M. W. Roth S. J. Luther E. Rose S. S. Springer T. A. (1994). Monocyte chemoattractant protein 1 acts as a T-lymphocyte chemoattractant. *Proc. Natl. Acad. Sci. U.S.A.* 91 3652–3656. 10.1073/pnas.91.9.3652 8170963 PMC43639

[B11] Chalmers J. D. Akram A. R. Singanayagam A. Wilcox M. H. Hill A. T. (2016). Risk factors for *Clostridium difficile* infection in hospitalized patients with community-acquired pneumonia. *J. Infect.* 73 45–53. 10.1016/j.jinf.2016.04.008 27105657

[B12] Chao A. Bunge J. (2002). Estimating the number of species in a stochastic abundance model. *Biometrics* 58 531–539. 10.1111/j.0006-341X.2002.00531.x 12229987

[B13] Chen X. Katchar K. Goldsmith J. D. Nanthakumar N. Cheknis A. Gerding D. N. (2008). A mouse model of *Clostridium difficile*-associated disease. *Gastroenterology* 135 1984–1992. 10.1053/j.gastro.2008.09.002 18848941

[B14] Chen Y. B. Gu S. L. Wei Z. Q. Shen P. Kong H. S. Yang Q. (2014). Molecular epidemiology of *Clostridium difficile* in a tertiary hospital of China. *J. Med. Microbiol.* 63(Pt 4) 562–569. 10.1099/jmm.0.068668-0 24344206

[B15] Chitnis A. S. Holzbauer S. M. Belflower R. M. Winston L. G. Bamberg W. M. Lyons C. (2013). Epidemiology of community-associated *Clostridium difficile* infection, 2009 through 2011. *JAMA Intern. Med.* 173 1359–1367. 10.1001/jamainternmed.2013.7056 23780507 PMC11931991

[B16] Cornely O. A. Crook D. W. Esposito R. Poirier A. Somero M. S. Weiss K. (2012). Fidaxomicin versus vancomycin for infection with *Clostridium difficile* in Europe, Canada, and the USA: a double-blind, non-inferiority, randomised controlled trial. *Lancet Infect. Dis.* 12 281–289. 10.1016/S1473-3099(11)70374-7 22321770

[B17] Cowardin C. A. Jackman B. M. Noor Z. Burgess S. L. Feig A. L. Petri W. A. Jr. (2016). Glucosylation drives the innate inflammatory response to *Clostridium difficile* toxin A. *Infect. Immun.* 84 2317–2323. 10.1128/IAI.00327-16 27271747 PMC4962640

[B18] Di Bella S. Ascenzi P. Siarakas S. Petrosillo N. di Masi A. (2016). *Clostridium difficile* toxins A and B: insights into pathogenic properties and extraintestinal effects. *Toxins* 8:E134. 10.3390/toxins8050134 27153087 PMC4885049

[B19] Edgar R. C. (2013). UPARSE: highly accurate OTU sequences from microbial amplicon reads. *Nat. Methods* 10 996–998. 10.1038/nmeth.2604 23955772

[B20] Gebhart D. Lok S. Clare S. Tomas M. Stares M. Scholl D. (2015). A modified R-type bacteriocin specifically targeting *Clostridium difficile* prevents colonization of mice without affecting gut microbiota diversity. *mBio* 6:e2368. 10.1128/mBio.02368-14 25805733 PMC4453579

[B21] Goldenberg J. Z. Lytvyn L. Steurich J. Parkin P. Mahant S. Johnston B. C. (2015). Probiotics for the prevention of pediatric antibiotic-associated diarrhea. *Cochrane Database Syst. Rev.* 18:CD004827. 10.1002/14651858.CD004827.pub4 26695080

[B22] Gu S. Chen Y. Zhang X. Lu H. Lv T. Shen P. (2016). Identification of key taxa that favor intestinal colonization of *Clostridium difficile* in an adult Chinese population. *Microbes Infect.* 18 30–38. 10.1016/j.micinf.2015.09.008 26383014

[B23] Kanehisa M. Goto S. Sato Y. Furumichi M. Tanabe M. (2012). KEGG for integration and interpretation of large-scale molecular data sets. *Nucleic Acids Res.* 40 D109–D114. 10.1093/nar/gkr988 22080510 PMC3245020

[B24] Kim J. M. Lee J. Y. Yoon Y. M. Oh Y. K. Youn J. Kim Y. J. (2006). NF-kappa B activation pathway is essential for the chemokine expression in intestinal epithelial cells stimulated with *Clostridium difficile* toxin A. *Scand. J. Immunol.* 63 453–460. 10.1111/j.1365-3083.2006.001756.x 16764699

[B25] Koon H. W. Su B. Xu C. Mussatto C. C. Tran D. H. Lee E. C. (2016). Probiotic Saccharomyces boulardii CNCM I-745 prevents outbreak-associated *Clostridium difficile*-associated cecal inflammation in hamsters. *Am. J. Physiol. Gastrointest. Liver Physiol.* 311 G610–G623. 10.1152/ajpgi.00150.2016 27514478 PMC5142203

[B26] Kostova Z. Batsalova T. Moten D. Teneva I. Dzhambazov B. (2015). Ragweed-allergic subjects have decreased serum levels of chemokines CCL2, CCL3, CCL4 and CCL5 out of the pollen season. *Cent. Eur. J. Immunol.* 40 442–446. 10.5114/ceji.2015.56965 26862308 PMC4737740

[B27] Langille M. G. Zaneveld J. Caporaso J. G. McDonald D. Knights D. Reyes J. A. (2013). Predictive functional profiling of microbial communities using 16S rRNA marker gene sequences. *Nat. Biotechnol.* 31 814–821. 10.1038/nbt.2676 23975157 PMC3819121

[B28] Leffler D. A. Lamont J. T. (2015). *Clostridium difficile* infection. *N. Engl. J. Med.* 372 1539–1548. 10.1056/NEJMra1403772 25875259

[B29] Lo Vecchio A. Zacur G. M. (2012). *Clostridium difficile* infection: an update on epidemiology, risk factors, and therapeutic options. *Curr. Opin. Gastroenterol.* 28 1–9. 10.1097/MOG.0b013e32834bc9a9 22134217

[B30] Lv L. X. Hu X. J. Qian G. R. Zhang H. Lu H. F. Zheng B. W. (2014a). Administration of *Lactobacillus salivarius* LI01 or *Pediococcus pentosaceus* LI05 improves acute liver injury induced by D-galactosamine in rats. *Appl. Microbiol. Biotechnol.* 98 5619–5632. 10.1007/s00253-014-5638-2 24639205

[B31] Lv L. X. Li Y. D. Hu X. J. Shi H. Y. Li L. J. (2014b). Whole-genome sequence assembly of *Pediococcus pentosaceus* LI05 (CGMCC 7049) from the human gastrointestinal tract and comparative analysis with representative sequences from three food-borne strains. *Gut Pathog.* 6:36. 10.1186/s13099-014-0036-y 25349631 PMC4209512

[B32] Magoc T. Salzberg S. L. (2011). FLASH: fast length adjustment of short reads to improve genome assemblies. *Bioinformatics* 27 2957–2963. 10.1093/bioinformatics/btr507 21903629 PMC3198573

[B33] Mehta H. M. Malandra M. Corey S. J. (2015). G-CSF and GM-CSF in neutropenia. *J. Immunol.* 195 1341–1349. 10.4049/jimmunol.1500861 26254266 PMC4741374

[B34] Mills J. P. Rao K. Young V. B. (2018). Probiotics for prevention of *Clostridium difficile* infection. *Curr. Opin. Gastroenterol.* 34 3–10. 10.1097/MOG.0000000000000410 29189354 PMC6335148

[B35] Ng J. Hirota S. A. Gross O. Li Y. Ulke-Lemee A. Potentier M. S. (2010). *Clostridium difficile* toxin-induced inflammation and intestinal injury are mediated by the inflammasome. *Gastroenterology* 139 542–552 552.e1–553.e1. 10.1053/j.gastro.2010.04.005 20398664

[B36] Nusrat A. von Eichel-Streiber C. Turner J. R. Verkade P. Madara J. L. Parkos C. A. (2001). *Clostridium difficile* toxins disrupt epithelial barrier function by altering membrane microdomain localization of tight junction proteins. *Infect. Immun.* 69 1329–1336. 10.1128/IAI.69.3.1329-1336.2001 11179295 PMC98024

[B37] O’Connor J. R. Johnson S. Gerding D. N. (2009). *Clostridium difficile* infection caused by the epidemic BI/NAP1/027 strain. *Gastroenterology* 136 1913–1924. 10.1053/j.gastro.2009.02.073 19457419

[B38] Pawlowski S. W. Calabrese G. Kolling G. L. Platts-Mills J. Freire R. AlcantaraWarren C. (2010). Murine model of *Clostridium difficile* infection with aged gnotobiotic C57BL/6 mice and a BI/NAP1 strain. *J. Infect. Dis.* 202 1708–1712. 10.1086/657086 20977342 PMC3057484

[B39] Pruitt R. N. Chambers M. G. Ng K. K. Ohi M. D. Lacy D. B. (2010). Structural organization of the functional domains of *Clostridium difficile* toxins A and B. *Proc. Natl. Acad. Sci. U.S.A.* 107 13467–13472. 10.1073/pnas.1002199107 20624955 PMC2922184

[B40] Quigley E. M. Stanton C. Murphy E. F. (2013). The gut microbiota and the liver. Pathophysiological and clinical implications. *J. Hepatol.* 58 1020–1027. 10.1016/j.jhep.2012.11.023 23183530

[B41] Ratsep M. Koljalg S. Sepp E. Smidt I. Truusalu K. Songisepp E. (2017). A combination of the probiotic and prebiotic product can prevent the germination of *Clostridium difficile* spores and infection. *Anaerobe* 47 94–103. 10.1016/j.anaerobe.2017.03.019 28465256

[B42] Roychowdhury S. Cadnum J. Glueck B. Obrenovich M. Donskey C. Cresci G. A. M. (2018). *Faecalibacterium prausnitzii* and a prebiotic protect intestinal health in a mouse model of antibiotic and *Clostridium difficile* exposure. *JPEN J. Parenter. Enteral Nutr.* 42 1156–1167. 10.1002/jpen.1053 29385239 PMC6068000

[B43] Schaffler H. Breitruck A. (2018). *Clostridium difficile* - from colonization to infection. *Front. Microbiol.* 9:646 10.3389/fmicb.2018.00646 PMC5902504 29692762

[B44] Schenck L. P. Hirota S. A. Hirota C. L. Boasquevisque P. Tulk S. E. Li Y. (2013). Attenuation of *Clostridium difficile* toxin-induced damage to epithelial barrier by ecto-5’-nucleotidase (CD73) and adenosine receptor signaling. *Neurogastroenterol. Motil.* 25 e441–e453. 10.1111/nmo.12139 23600886

[B45] Shen N. T. Maw A. Tmanova L. L. Pino A. Ancy K. Crawford C. V. (2017). Timely use of probiotics in hospitalized adults prevents *Clostridium difficile* infection: a systematic review with meta-regression analysis. *Gastroenterology* 152 1889.e9–1900.e9. 10.1053/j.gastro.2017.02.003 28192108

[B46] Shi D. Lv L. Fang D. Wu W. Hu C. Xu L. (2017). Administration of *Lactobacillus salivarius* LI01 or *Pediococcus pentosaceus* LI05 prevents CCl4-induced liver cirrhosis by protecting the intestinal barrier in rats. *Sci. Rep.* 7:6927. 10.1038/s41598-017-07091-1 28761060 PMC5537250

[B47] Spinler J. K. Auchtung J. Brown A. Boonma P. Oezguen N. Ross C. L. (2017). Next-generation probiotics targeting *Clostridium difficile* through precursor-directed antimicrobial biosynthesis. *Infect. Immun.* 85 e303–e317. 10.1128/IAI.00303-17 28760934 PMC5607411

[B48] Theriot C. M. Bowman A. A. Young V. B. (2016). Antibiotic-induced alterations of the gut microbiota alter secondary bile acid production and allow for *Clostridium difficile* spore germination and outgrowth in the large intestine. *mSphere* 1:e00045-15. 10.1128/mSphere.00045-15 27239562 PMC4863611

[B49] Theriot C. M. Koenigsknecht M. J. Carlson P. E. Jr Hatton G. E. Nelson A. M. Li B. (2014). Antibiotic-induced shifts in the mouse gut microbiome and metabolome increase susceptibility to *Clostridium difficile* infection. *Nat. Commun.* 5:3114. 10.1038/ncomms4114 24445449 PMC3950275

[B50] Valdes-Varela L. Gueimonde M. Ruas-Madiedo P. (2018). Probiotics for prevention and treatment of *Clostridium difficile* infection. *Adv. Exp. Med. Biol.* 1050 161–176. 10.1007/978-3-319-72799-8_10 29383669

[B51] Vanderhoof J. A. Whitney D. B. Antonson D. L. Hanner T. L. Lupo J. V. Young R. J. (1999). *Lactobacillus* GG in the prevention of antibiotic-associated diarrhea in children. *J. Pediatr.* 135 564–568. 10.1016/S0022-3476(99)70053-3 10547243

[B52] Vohra P. Poxton I. R. (2012). Induction of cytokines in a macrophage cell line by proteins of *Clostridium difficile*. *FEMS Immunol. Med. Microbiol.* 65 96–104. 10.1111/j.1574-695X.2012.00952.x 22409477

[B53] von Eichel-Streiber C. Laufenberg-Feldmann R. Sartingen S. Schulze J. Sauerborn M. (1992). Comparative sequence analysis of the *Clostridium difficile* toxins A and B. *Mol. Gen. Genet.* 233 260–268. 10.1007/BF00587587 1603068

[B54] Wang Q. Garrity G. M. Tiedje J. M. Cole J. R. (2007). Naive Bayesian classifier for rapid assignment of rRNA sequences into the new bacterial taxonomy. *Appl. Environ. Microbiol.* 73 5261–5267. 10.1128/AEM.00062-07 17586664 PMC1950982

[B55] Xu Q. Chen Y. Gu S. Lv T. Zheng B. Shen P. (2017). Hospital-acquired *Clostridium difficile* infection in Mainland China: a seven-year (2009-2016) retrospective study in a large university hospital. *Sci. Rep.* 7:9645. 10.1038/s41598-017-09961-0 28852010 PMC5575102

