# Supplementary figures and images for: Protective Effect of Pediococcus pentosaceus LI05 Against Clostridium difficile Infection in a Mouse Model
Source: Front Microbiol. 2018 Oct 9;9:2396. doi: 10.3389/fmicb.2018.02396 (PMC6189400; doi:10.3389/fmicb.2018.02396)

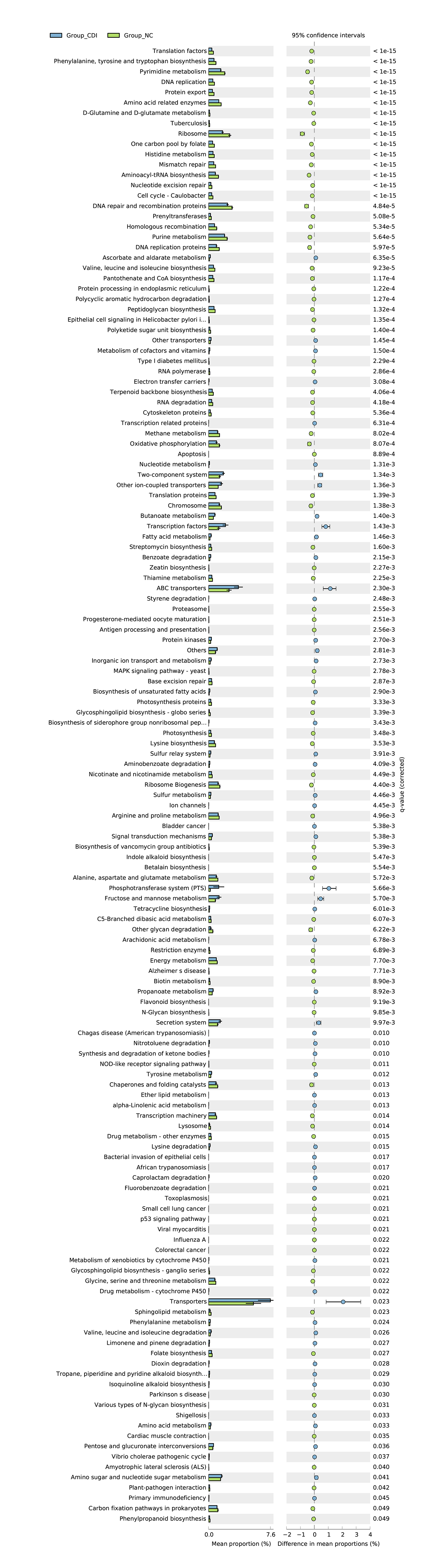

Supplement: FIGURE S1 — PICRUSt results of metabolic pathways in the gut microbiome of CDI group and NC group. [file Image_1.TIF]
