# Supplementary material for: Protective Effect of Pediococcus pentosaceus LI05 Against Clostridium difficile Infection in a Mouse Model
Source: Front Microbiol. 2018 Oct 9;9:2396. doi: 10.3389/fmicb.2018.02396 (PMC6189400; doi:10.3389/fmicb.2018.02396)
Supplement: TABLE S1 — PCR primer sets for RT-PCR analysis of the indicated genes. [file Table_1.DOCX]

Supplementary Material

Protective Effect of Pediococcus pentosaceus LI05 against Clostridium difficile infection in a mouse model

**Qiaomai Xu**^a^**, Silan Gu**^a^**, Yunbo Chen**^a^**, Jiazheng Quan, Longxian Lv, Dazhi Chen, Beiwen zheng, Lichen Xu and Lanjuan Li^*^**

*** Correspondence:** Lanjuan Li: [ljli@zju.edu.cn](mailto:ljli@zju.edu.cn)

# Supplementary Figures and Tables

For more information on Supplementary Material and for details on the different file types accepted, please see [here](http://home.frontiersin.org/about/author-guidelines#SupplementaryMaterial).

## Supplementary Tables

| **Supplementary Table. 1. PCR primer sets for RT-PCR analysis of the indicated genes** | | |
| --- | --- | --- |
| Gene | Forward Sequence (5'-3') | Reverse Sequence (5'-3') |
| TNF-𝛼 | CAGGCGGTGCCTATGTCTC | CGATCACCCCGAAGTTCAGTAG |
| MCP-1 | CTGAGTTGACTCCTACTGTGGA | TCTTCCCAGGGTCGATAAAGT |
| MIP-1𝛼 | TGTACCATGACACTCTGCAAC | CAACGATGAATTGGCGTGGAA |
| ZO-1 | GCCGCTAAGAGCACAGCAA | GCCCTCCTTTTAACACATCAGA |
| occluding | TGAAAGTCCACCTCCTTACAGA | CCGGATAAAAAGAGTACGCTGG |
| claudin-1 | TGCCCCAGTGGAAGATTTACT | CTTTGCGAAACGCAGGACAT |
| Β-actin | GTGACGTTGACATCCGTAAAGA | GCCGGACTCATCGTACTCC |

**
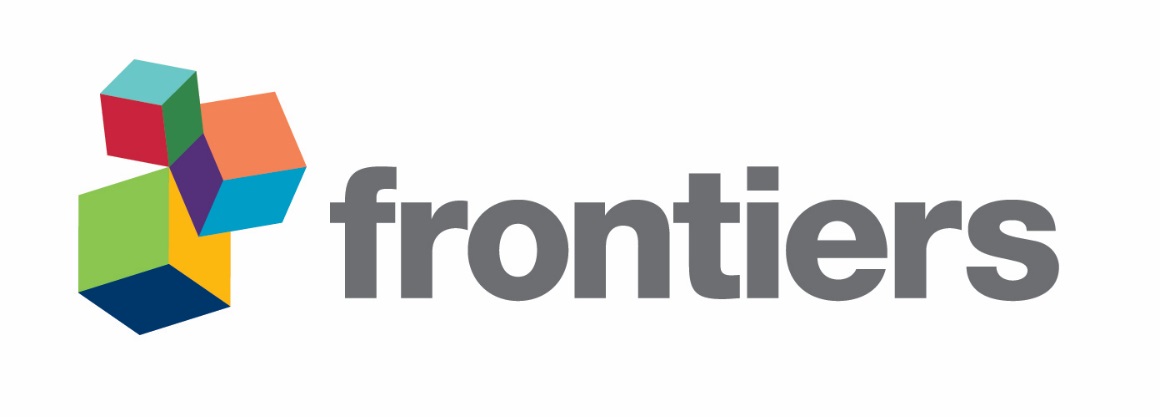
**
